# From pathogenesis to treatment: the impact of bacteria on cancer

**DOI:** 10.3389/fmicb.2024.1462749

**Published:** 2024-09-18

**Authors:** Jiatong Lu, Qiang Tong

**Affiliations:** Department of Gastrointestinal Surgery I Section, Renmin Hospital of Wuhan University, Wuhan, China

**Keywords:** cancer, bacteria, tumorigenesis, tumor prediction, therapeutic strategies

## Abstract

The intricate relationship between cancer and bacteria has garnered increasing attention in recent years. While traditional cancer research has primarily focused on tumor cells and genetic mutations, emerging evidence highlights the significant role of microbial communities within the tumor microenvironment in cancer development and progression. This review aims to provide a comprehensive overview of the current understanding of the complex interplay between cancer and bacteria. We explore the diverse ways in which bacteria influence tumorigenesis and tumor behavior, discussing direct interactions between bacteria and tumor cells, their impact on tumor immunity, and the potential modulation of the tumor microenvironment. Additionally, we delve into the mechanisms through which bacterial metabolites and extracellular products May affect cancer pathways. By conducting a thorough analysis of the existing literature, we underscore the multifaceted and intricate relationship between bacteria and cancer. Understanding this complex interplay could pave the way for novel therapeutic approaches and preventive strategies in cancer treatment.

## 1 Introduction

As one of the most deadly and complex diseases globally, cancer has long captivated the attention of scientists, doctors, and researchers. Despite significant advancements in cancer treatment and prevention over the past few decades, the precise etiology and underlying mechanisms of cancer development remain largely enigmatic. Recently, an increasing body of research has begun to underscore the potential role of microbes, particularly bacteria, in the initiation and progression of cancer (Sepich-Poore et al., 2021). This burgeoning field presents a novel perspective on cancer pathogenesis and holds the potential to revolutionize therapeutic strategies, offering new avenues for treatment.

Traditionally, cancer research has primarily centered on the abnormal proliferation and genetic mutations of tumor cells. However, with advancements in technology and the broadening of scientific perspectives, accumulating evidence now indicates that bacteria are integral players in tumor development (Yang et al., 2023; Goto, 2022; Ghaddar et al., 2022). The relationship between bacteria and tumors is complex and multifaceted. Bacteria can directly influence tumor cell proliferation, invasion, and metastasis through direct interactions. Additionally, bacteria have the capacity to modulate the immune response within the tumor microenvironment, potentially compromising the host’s immune defense against cancer and facilitating immune evasion by the tumor. Furthermore, bacterial metabolites and extracellular products can exert significant effects on tumor cells, further contributing to cancer progression.

Despite the growing body of evidence supporting the role of bacteria in tumor development, our understanding of the intricate interplay between bacteria and cancer remains limited. Research in this domain is still in its early stages, with many critical questions yet to be addressed. A comprehensive understanding of the presence and mechanisms of bacterial influence across various cancer types, as well as their complex interactions with the host immune system, tumor cells, and diverse microbial communities, is essential for advancing our knowledge in this field. This review seeks to provide a detailed examination and synthesis of current research advancements regarding the complex association between bacteria and cancer, and to explore the potential implications of these findings for novel cancer treatment and prevention strategies.

## 2 The role of bacteria in cancer development and prediction

### 2.1 Intracellular bacteria

**Figure 1:**
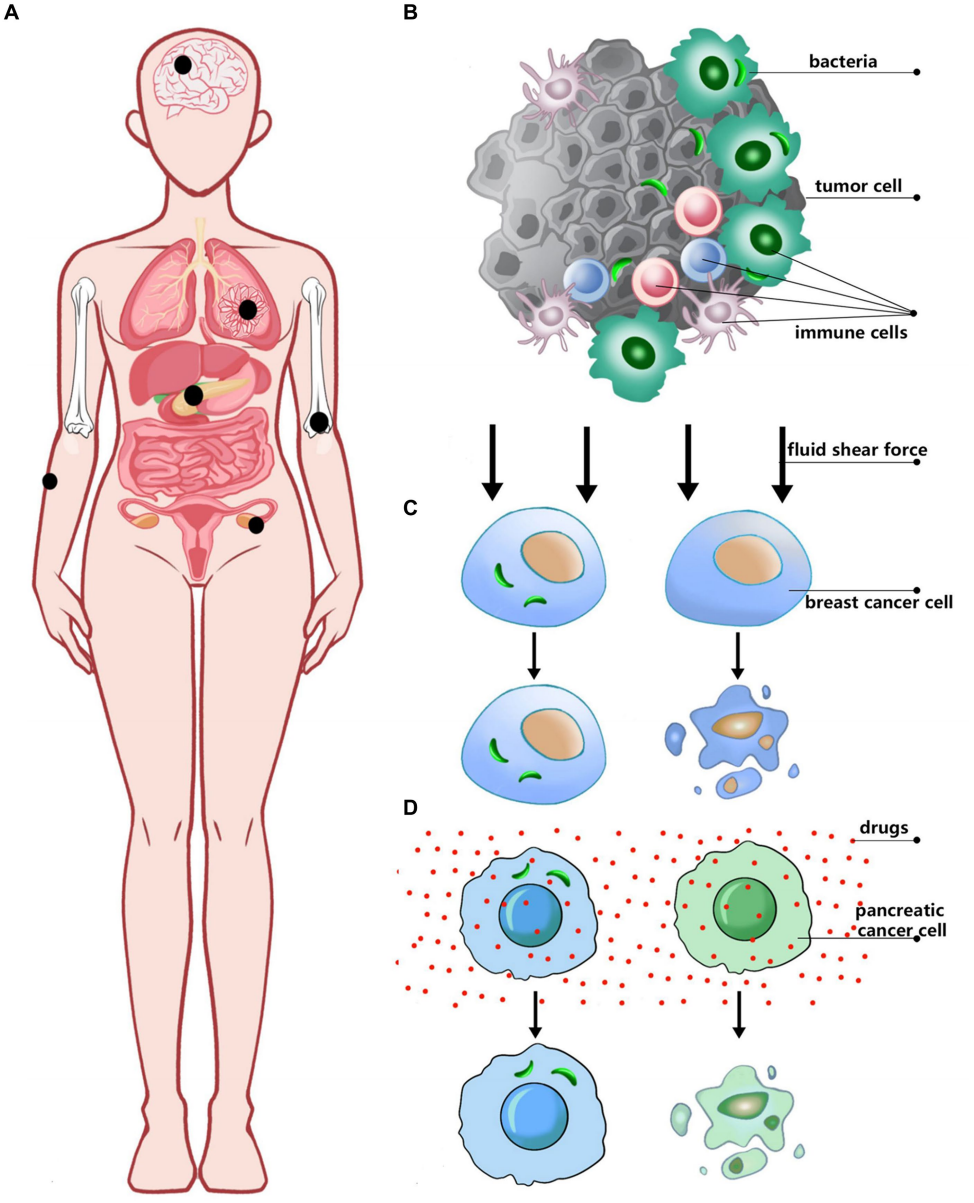
The relationship between intracellular bacteria and tumors. **(A)** Tumor types with intracellular bacteria have been discovered. **(B)** The location of bacteria in tumor tissue. **(C,D)** The role of bacteria in different cancers.

The traditional belief is that there are no bacteria in tumor tissue, but with the advancement of research, it has been demonstrated that bacteria primarily exist in tumor cells and immune cells in tumor tissues (Nejman et al., 2020; Livyatan et al., 2020) by using 16srDNA sequencing, QPCR, FISH, LPS, and LTA antibody fluorescence staining (Fu et al., 2022; Geller et al., 2017) and other experimental techniques (Figure 1).

Although these approaches May have limitations, including potential contamination, sensitivity to detection, and difficulty in distinguishing between live bacteria and bacterial residues, a growing body of research has shown that a wide variety of bacteria are present in different types of tumors, and that these bacteria influence tumor progression through different mechanisms. In breast cancer cells, intracellular bacteria are relatively diverse, primarily originating from three phyla: Proteobacteria, Firmicutes, and Actinobacteria. Although *Staphylococcus*, *Lactobacillus*, *Streptococcus*, and *Enterococcus* are present at lower abundances, they play a crucial role in modulating the RhoA/ROCK signaling pathway. This modulation reduces intracellular mechanical stress, thereby enhancing the resistance of circulating tumor cells to fluid shear stress. As a result, the survival of these tumor cells in the bloodstream is improved, facilitating their metastatic colonization in distant organs (Nejman et al., 2020; Fu et al., 2022). Intracellular bacteria have also been identified within pancreatic cancer cells, predominantly from the phylum Proteobacteria, including the families Enterobacteriaceae and Pseudomonadaceae. These bacteria are believed to translocate retrogradely from the duodenum into the pancreas. Notably, *Mycoplasma hyorhinis*, a small, cell wall-deficient bacterium, has been detected not only in normal human dermal fibroblasts but also in mouse models of colorectal cancer and in human pancreatic ductal adenocarcinoma (PDAC) tissues. *Mycoplasma hyorhinis* confers chemoresistance to cancer cells by metabolizing gemcitabine into its inactive metabolite, 2′,2′-difluorodeoxyuridine, through its cytidine deaminase (CDD) activity. Additionally, other bacteria within the Enterobacteriaceae family May contribute to tumor resistance by modulating drug concentrations within the tumor microenvironment, thereby influencing tumor growth and metastasis (Geller et al., 2017).

Intracellular bacteria have also been identified in various cancers, including melanoma, ovarian cancer, bone tumors, and glioblastoma multiforme. In particular, Firmicutes are notably abundant in ovarian cancer, while Actinobacteria play a significant role in non-gastrointestinal tumors. In melanoma, the detected bacteria are predominantly Gram-positive (Nejman et al., 2020) and have been shown to stimulate the body’s immune system to target and eliminate tumor cells through peptide presentation (Sepich-Poore et al., 2021). Understanding the presence and specific roles of these bacteria within tumor tissues could pave the way for novel therapeutic approaches, further illuminating the complex and multifaceted relationship between bacteria and cancer.

### 2.2 Non-intracellular bacteria

Extracellular bacteria also play a significant role in the onset and progression of tumors. Table 1 provides an overview of the distribution and functions of several common bacteria associated with tumorigenesis.

**Table 1:** Cancer related microorganisms.

Microorganism	Phylum	Natural habitat	Characteristics in cancer	Related cancer	References
*Staphylococcus aureus*	Firmicutes	Skin, mucosa, nasal cavity, oral cavity	Being specific for tumorogenic tissues	Oral cancers	Chocolatewala et al. (2010)
*Veillonella parvula*	Firmicutes	Oral cavity	Being specific for tumorogenic tissues	Oral cancers	Chocolatewala et al. (2010)
*Prevotella melaninogenica*	Bacteroidetes	Oral cavity, respiratory tract	Being specific for tumorogenic tissues	Oral cancers	Chocolatewala et al. (2010)
*S. anginosus*	Firmicutes	Oral cavity, throat	Significant risk factors associated with both esophageal cancer and oral squamous cell carcinoma	Esophageal cancer and oral squamous cell carcinoma	Xiao et al. (2020), Kawasaki et al. (2021), Moghimi et al. (2020)
*A. actinomycetemcomitans*	Proteobacteria	Oral cavity	Significant risk factors associated with both esophageal cancer and oral squamous cell carcinoma	Esophageal cancer and oral squamous cell carcinoma	Xiao et al. (2020), Kawasaki et al. (2021), Moghimi et al. (2020)
*T. forsythia*	Bacteroidetes	Oral cavity (associated with periodontal disease)	Significant risk factors associated with both esophageal cancer and oral squamous cell carcinoma	Esophageal cancer and oral squamous cell carcinoma	Xiao et al. (2020), Kawasaki et al. (2021), Moghimi et al. (2020)
*Tannerella forsythia*	Bacteroidetes	Oral cavity (associated with periodontal disease)	Had the peptidyl arginine deaminase (PAD), which may be one of the causes of the P53 gene mutation	Pancreatic cancer	Öğrendik (2015), Michaud et al. (2013)
*Porphyromonas gingivalis*	Bacteroidetes	Oral cavity (associated with periodontal disease)	Had the peptidyl arginine deaminase (PAD), which May be one of the causes of the P53 gene mutation	Pancreatic cancer	Öğrendik (2015), Michaud et al. (2013)
*Prevotella intermedia*	Bacteroidetes	Oral cavity	Had the peptidyl arginine deaminase (PAD), which may be one of the causes of the P53 gene mutation	Pancreatic cancer	Öğrendik (2015), Michaud et al. (2013)
*Treponema denticola*	Spirochaetes	Oral cavity (associated with periodontal disease)	Had the peptidyl arginine deaminase (PAD), which May be one of the causes of the P53 gene mutation	Pancreatic cancer	Öğrendik (2015), Michaud et al. (2013)
*Helicobacter pylori*	Proteobacteria	Stomach	Secreting a significant number of inflammatory mediators; increasing the level of oxidative stress in cells, silence anti-tumor miRNAs by regulating DNA methylation at the upstream of miRNA, histone post-transcriptional modification, DNA damage, and repair	Gastric cancer, pancreatic cancer and pancreatic autoimmune inflammation	Zou et al. (2019), Qu et al. (2013), Ferreira et al. (2018), Gunathilake et al. (2021), Li and Yu (2020), Li and Perez Perez (2018)
*Prevotella intermedius*	Bacteroidetes	Oral cavity, possibly the gut	Increased bacterial abundance in gastric cancer patients	Gastric cancer	Ferreira et al. (2018)
*Neisseria gonorrhoeae*	Proteobacteria	Genital tract	Increased bacterial abundance in gastric cancer patients	Gastric cancer	Ferreira et al. (2018)
*Streptococcus*	Firmicutes	Oral cavity, throat, gut	Increased bacterial abundance in gastric cancer patients	Gastric cancer	Ferreira et al. (2018)
*Ruminococcus gnavus*	Firmicutes	Gut	Enriched in normal tissues, inhibits CD8+ T cells activity	Colorectal cancer	Zhang et al. (2023)
*Blautia producta*	Firmicutes	Gut	Enriched in normal tissues, inhibits CD8+ T cells activity	Colorectal cancer	Zhang et al. (2023)
*Dorea formicigenerans*	Firmicutes	Gut	Enriched in normal tissues, inhibits CD8+ T cells activity	Colorectal cancer	Zhang et al. (2023)
*Bacteroides fragilis*	Bacteroidetes	Gut	Modulating the host’s metabolic pathways, immune responses, and inflammatory reactions, influencing host signaling pathways	Inflammatory bowel disease (IBD) and colorectal cancer	Chen et al. (2024), Santaolalla et al. (2013), Wu et al. (2004), Goodwin et al. (2011), Thiele Orberg et al. (2017), Wu et al. (1998)
*Peptostreptococcus anaerobius*	Firmicutes	Gut, skin, mucous membranes	Modulating the host’s metabolic pathways, immune responses, and inflammatory reactions, influencing host signaling pathways	Inflammatory bowel disease and colorectal cancer	Chen et al. (2024), Santaolalla et al. (2013)
*Clostridium septicum*	Firmicutes	Gut, soil	Modulating the host’s metabolic pathways, immune responses, and inflammatory reactions, influencing host signaling pathways	Inflammatory bowel disease and colorectal cancer	Chen et al. (2024), Santaolalla et al. (2013)
*Parvimonas micra*	Firmicutes	Oral cavity, gut	Modulating the host’s metabolic pathways, immune responses, and inflammatory reactions, influencing host signaling pathways	Inflammatory bowel disease and colorectal cancer	Chen et al. (2024), Santaolalla et al. (2013)
*Fusobacterium nucleatum*	Fusobacteria	Oral cavity, gut,	Modulating the host’s metabolic pathways, immune responses, and inflammatory reactions, influencing host signaling pathways; Induces cervical cancer progression through cytotoxic effects, immune regulation, and lipid metabolism interference	colorectal cancer; cervical cancer	Shi et al. (2016), Kitajima and Barbie (2018), Mohammadi et al. (2022), Maarsingh et al. (2022)
*Prevotella*	Bacteroidetes	Oral cavity, gut, urogenital tract	Enriched in endometrial cancer; associated with gene regulation (PRSS33, CPB2, XBP1) involved in fibrin degradation and cell secretion; Increased abundance linked to inflammation and lung tissue changes, contributing to NSCLC progression	Endometrial cancer, non-small cell lung cancer	Li et al. (2021), Qian et al. (2022)
*Pelomonas*	Proteobacteria	Various environments, including water	Promoting the development of endometrial cancer	Endometrial cancer, bacterial vaginosis	Li et al. (2021)
*Lancefieldella parvula*	Firmicutes	Urogenital tract	Induces cervical cancer progression through cytotoxic effects, immune regulation, and lipid metabolism interference	Cervical cancer	Maarsingh et al. (2022)
*Fusobacterium gonidiaformans*	Fusobacteria	Oral cavity, urogenital tract	Induces cervical cancer progression through cytotoxic effects, immune regulation, and lipid metabolism interference	Cervical cancer	Maarsingh et al. (2022)
*Peptoniphilus lacrimalis*	Firmicutes	Urogenital tract	Induces cervical cancer progression through cytotoxic effects, immune regulation, and lipid metabolism interference	Cervical cancer	Maarsingh et al. (2022)
*Porphyromonas uenonis*	Bacteroidetes	Oral cavity, urogenital tract	Induces cervical cancer progression through cytotoxic effects, immune regulation, and lipid metabolism interference	Cervical cancer	Maarsingh et al. (2022)
*Escherichia coli*	Proteobacteria	Gut, urogenital tract	Enhances expression of lipid synthesis enzymes (FASN, ACC1) via TLR4/TLR9 pathways, promoting proliferation and metastasis	Non-small cell lung cancer	Ye et al. (2016)
*Roseburia*	Firmicutes	Gut	Altered abundance associated with lung tissue changes, contributing to NSCLC progression	Non-small cell lung cancer	Qian et al. (2022)
*Gemmiger*	Firmicutes	Gut	Altered abundance associated with lung tissue changes, contributing to NSCLC progression	Non-small cell lung cancer	Qian et al. (2022)

#### 2.2.1 Oral bacteria

Many studies of the role of oral bacteria in cancer rely on observational data and microbiome analysis. While these methods provide valuable insights, they are subject to biases such as reverse causality and confounding factors, including diet and oral hygiene, that influence the composition of the microbiome. Despite these limitations, recent microbiological examinations have identified certain oral microorganisms that exhibit a notable selectivity for tumors, recent microbiological examinations of oral microorganisms from healthy volunteers and patients with oral cancer have revealed that certain bacteria, such as *Staphylococcus aureus*, *Exiguobacterium oxidotolerans*, *Veillonella parvula*, and *Prevotella melaninogenica* (Chocolatewala et al., 2010), exhibiting a notable selectivity for tumors, making them potential salivary markers for early oral cancer detection. Research indicates a positive correlation between oral bacteria and lung cancer (Zhou et al., 2022), while other studies highlight that bacteria present in dental plaque and saliva, particularly *S. anginosus*, *A. actinomycetemcomitans*, and *T. forsythia*, are significant risk factors for esophageal cancer and oral squamous cell carcinoma (Xiao et al., 2020; Kawasaki et al., 2021; Moghimi et al., 2020). Furthermore, bacteria in the root canal can promote cell proliferation and alter cancer cell biology, which May explain the carcinogenic potential of oral bacteria (Suprewicz et al., 2020).

Additionally, in conditions such as periodontitis, oral bacteria can enter the gut, disrupt the intestinal microflora, and cause an imbalance that triggers abnormal immune and inflammatory responses, ultimately leading to colorectal cancer (Ohashi et al., 2022; Koliarakis et al., 2019). Moreover, *Tannerella forsythia*, *Porphyromonas gingivalis*, *Prevotella intermedia*, and *Treponema denticola*, which possess peptidyl arginine deiminase, May contribute to P53 gene mutations in normal cells, thereby increasing the risk of pancreatic cancer (Öğrendik, 2015; Michaud et al., 2013), *P. gingivalis* can migrate from the mouth to the pancreas, and in wild-type mice, repeated administration of *P. gingivalis* induces acinar ductal metaplasia (ADM), which is considered a precursor of pancreatic intraepithelial neoplasia (PanIN). Further studies found that *P. gingivalis* also accelerated the progression of PanIN to pancreatic ductal adenocarcinoma (PDAC) by altering the composition of the pancreatic microbiota, under stress conditions, *P. gingivalis* can protect cancer cells from reactive oxygen species (ROS) -induced cell death, thus promoting the development of pancreatic cancer (Saba et al., 2024).

#### 2.2.2 Gastrointestinal bacteria

The human gastrointestinal system harbors approximately 100 trillion bacteria (Quazi, 2022), which serve a crucial part in maintaining normal physiological functions. Disruption of the intestinal microbiota can lead to the overproduction of harmful metabolites and toxins, which are associated with an increased risk of inflammation and cancer. In the stomach, *Helicobacter pylori* can cause gastric mucosal epithelial cells to secrete a significant number of inflammatory mediators, increase the level of oxidative stress in these cells, and ultimately cause cancer (Zou et al., 2019). *Helicobacter pylori* can silence anti-tumor miRNAs by regulating DNA methylation at the upstream of miRNA, histone post-transcriptional modification, DNA damage and repair, and finally induce carcinogenesis (Qu et al., 2013). These mechanisms establish *Helicobacter pylori* as a major carcinogen in gastric cancer. As gastric cancer progresses, the microbiota in patients also undergoes significant changes. 16srDNA sequencing results showed that the abundance of non-*H. pylori* bacteria (such a*s Proteobacteria*, *Firmicutes*, and *Actinomycetes*) was significantly increased in gastric cancer patients compared with healthy people. Although *Helicobacter pylori* remain the predominant bacterium in patients with chronic gastritis, its prevalence decreases as gastric cancer advances. Concurrently, other bacterial species, such as *Streptococcus*, *Prevotella intermedia*, and *Neisseria gonorrhoeae*, become significantly more abundant (Ferreira et al., 2018), this suggests that, in addition to *Helicobacter pylori*, other bacteria in the stomach May also contribute to the risk of developing gastric cancer (Gunathilake et al., 2021; Li and Yu, 2020; Li and Perez Perez, 2018), for instance, in mouse models, *Streptococcus anginosus* has been shown to promote the development and progression of gastric cancer by interacting with the Annexin A2 (ANXA2) receptor on gastric epithelial cells through its surface protein TMPC, this interaction enhances bacterial attachment and colonization, leading to the activation of the MAPK signaling pathway. Notably, the elimination of ANXA2 blocks the MAPK activation induced by *Streptococcus anginosus*, thereby inhibiting its tumorigenic effects (Fu et al., 2024). The involvement of these bacteria in the development of gastric cancer suggests their potential as biomarkers for predicting disease (Table 2). Studies have demonstrated that the analysis of tongue coating flora in healthy individuals and gastric cancer patients identified a combination of six bacterial genera—*Peptostreptococcus*, *Peptococcus*, *Porphyromonas*, *Macromonas*, *Rothia*, and *Fusobacterium*—as the most effective predictive model for distinguishing gastric cancer patients from healthy controls (Xu et al., 2021). Further microbiome analysis also revealed a significant increase in the abundance of *Oscillospira*, *Escherichia*, *Faecalibacterium*, and *Desulfovibrio* in the stool of gastric cancer patients. Notably, *Desulfovibrio* was significantly more abundant in stage IV gastric cancer patients compared to those in stages I, II, and III (Liu et al., 2021).

**Table 2:** The diagnostic potential of bacteria in different types of cancer.

Strain(s)	Type of cancer	Bacterial action	References
*P. melaninogenica* *Streptococcus mitis* *Capnocytophaga gingivalis*	Oral cancer	Distinct salivary specificity in oral cancer, making them potential salivary markers for early oral cancer detection	Chocolatewala et al. (2010)
*Parvimonas micra* *Clostridium symbiosis* *Hungatela hathewayi* *Peptostreptococcus stomatis* *Gemella morbillorum*	Colorectal cancer	Diagnostic model for patients with colorectal cancer	Zhang et al. (2022), Yao et al. (2021), Osman et al. (2021), Rezasoltani et al. (2018)
*Prevotella copri* *Parvimonas micra* *Parvimonas micra* *Cetobacterium somerae* *Gemella morbillorum*	Colorectal cancer	Diagnostic model for patients with colorectal cancer	Zhang et al. (2022), Yao et al. (2021), Osman et al. (2021), Rezasoltani et al. (2018)
*Enterococcus faecalis* *Streptococcus bovis* Enterotoxigenic *Bacteroides fragilis* *Porphyromonas* spp. *Fusobacterium nucleatum*	Colorectal cancer	Diagnostic model for patients with colorectal cancer	Zhang et al. (2022), Yao et al. (2021), Osman et al. (2021), Rezasoltani et al. (2018)
*Peptostreptococcou* *Peptococcus* *Porphyromonas* *Macromonas* *Rothia* *Fusobacterium*	Gastric cancer	Prediction model for distinguishing gastric cancer patients from healthy people	Xu et al. (2021), Liu et al. (2021)
*Oscillospira* *Escherichia* *Faecalibacterium* *Desulfovibrio*	Gastric cancer	Potential biomarkers for predicting gastric cancer	Xu et al. (2021), Liu et al. (2021)

Intestinal bacteria are closely linked to the pathogenesis of inflammatory bowel disease (IBD) and colorectal cancer (CRC). Studies have revealed significant differences in the composition of intestinal flora among healthy individuals, IBD patients, and CRC patients (Ma et al., 2021), compared to normal individuals and CRC patients, IBD patients exhibit an increased abundance of *Bacteroides* and a decreased abundance of Firmicutes, in contrast, CRC patients demonstrate an increased abundance of Fusobacteria, Firmicutes, Verrucomicrobia, Bacteroides, and Proteobacteria (Xu et al., 2020). Further studies showed that symbiotic bacteria belonging to the Lachnospiraceae family, such as *Ruminococcus gnavus*, *Blautia producta*, and *Dorea formicigenerans*, are enriched in normal tissues, they can degrade glycerolysin, a compound that inhibits CD8(+) T cell activity. By breaking down glycerolysin, these bacteria promote the activation of CD8(+) T cells, thereby enhancing immune surveillance and inhibiting the growth of colon tumors (Zhang et al., 2023); *Fusobacterium nucleatum* and *Peptostreptococcus anaerobius* were more common in tumor tissues, among them, *Fusobacterium nucleatum* plays a multifaceted role in colorectal cancer progression. The FadA protein secreted by *Fusobacterium nucleatum* has been shown to regulate epithelial cell proliferation, contributing to tumor growth (Taddese et al., 2020). Additionally, *Fusobacterium nucleatum*-derived outer membrane vesicles (OMVs) can also significantly enhance the metastatic potential of cancer cells, specifically, in mouse models, these OMVs promote lung metastasis and increase cancer cell migration and invasion *in vitro*, the underlying mechanisms include the activation of autophagy flux and alterations in the expression of proteins associated with epithelial-mesenchymal transition (EMT) (Chen et al., 2024). Moreover, *Fusobacterium nucleatum*’s outer membrane protein contains lipopolysaccharide (LPS), a pathogen-associated molecular pattern that binds to Toll-like receptor 4 (TLR4) on the surface of host cells. This interaction initiates the TLR4 signaling pathway, leading to the activation of the myeloid differentiation primary response gene 88 (MYD88), a crucial adaptor protein that triggers the downstream NF-κB pathway. Once activated, NF-κB translocates to the nucleus, where it promotes the expression of pro-inflammatory cytokines and tumor-promoting genes such as miR-21. MiR-21, a well-known pro-inflammatory microRNA associated with colorectal cancer, inhibits the expression of RAS-GTPase activating protein family member RASA1, resulting in the sustained activation of the RAS signaling pathway and the subsequent initiation and progression of colorectal cancer (Santaolalla et al., 2013; Shi et al., 2016; Kitajima and Barbie, 2018).

Anaerobic bacteria like *Bacteroides fragilis*, *Peptostreptococcus anaerobius*, *Clostridium septicum*, *Porphyromonas gingivalis*, and *Parvimonas micra* play pivotal roles in colorectal cancer progression by modulating the host’s metabolic pathways, immune responses, and inflammatory reactions (Mohammadi et al., 2022; Justesen et al., 2022). *Bacteroides fragilis*, for instance, secretes a zinc-dependent metalloprotease known as *Bacteroides fragilis* toxin (BFT), which degrades E-cadherin in epithelial cells, leading to the nuclear translocation of *β*-catenin and activation of the Wnt/β-catenin signaling pathway. Concurrently, BFT activates the p38 MAPK and NF-κB signaling pathways. Activation of p38 MAPK upregulates the expression of cyclooxygenase-2 and prostaglandin E2, both of which play critical roles in cancer cell proliferation and tumor formation. Moreover, p38 MAPK regulates Spermine oxidase (SMO), resulting in the production of reactive oxygen species, which cause DNA damage and increased cell proliferation (Wu et al., 2004; Goodwin et al., 2011). Furthermore, BFT enhances the expression of CXC chemokines via the NF-κB pathway, promoting the recruitment of inflammatory cells and sustaining cancer cell proliferation within CRC tissues (Thiele Orberg et al., 2017; Wu et al., 1998). The significant role these bacteria play in CRC progression also highlights their potential as biomarkers for cancer prediction (Table 2). For example, studies have shown that the abundance of bacteria such as *Parvimonas micra*, *Clostridium symbiosum*, *Hungatella hathewayi*, *Peptostreptococcus stomatis*, and *Gemella morbillorum* increases significantly in CRC patients, suggesting that a combination of these bacteria could serve as a diagnostic model for CRC (Zhang et al., 2022). Similarly, another study proposed a prediction model based on the presence of *Prevotella copri*, *Parvimonas micra*, *Cetobacterium somerae*, and *Gemella morbillorum* (Yao et al., 2021). In addition, *Fusobacterium nucleatum*, *Akkermansia muciniphila*, *Parvimonas micra*, and *Peptostreptococcus stomatis* have also been detected in a large number of CRC patients (Osman et al., 2021). The combination of *Enterococcus faecalis*, *Streptococcus bovis*, *Bacteroides fragilis*, *Porphyromonas* spp. and *Fusobacterium nucleatum* also showed a high diagnostic value for CRC (Rezasoltani et al., 2018).

Certain bacterial metabolites, such as butyrate, have been shown to have anti-inflammatory and anticancer effects, and can enhance the efficacy of chemotherapy drugs (such as gemcitabine) against pancreatic cancer (Tarashi et al., 2019; Panebianco et al., 2022). Butyrate inhibits the development of colorectal cancer by maintaining intestinal epithelial barrier function, inhibiting inflammation, and inducing cancer cell apoptosis. It also acts as a histone deacetylase (HDAC) inhibitor, regulating gene expression and thereby preventing the proliferation and migration of cancer cells. However, some *Porphyromonas* species also secrete butyrate, but accelerate the development of colorectal cancer by inducing cell senescence (Qu et al., 2023), this finding suggests that the same metabolite, butyrate, May play complex and context-dependent roles in cancer development depending on its bacterial source, the imbalance of butyrate levels is also shown in patients with non-small cell lung cancer, indicating that metabolites produced by intestinal flora play an important role in the occurrence and progression of cancer (Gui et al., 2020). Beyond butyrate, other bacterial metabolites also influence tumor progression. For example, indole-3-propionic acid (IPA), a metabolite derived from *Lactobacillus johnsonii* or tryptophan, can enhance the efficacy of *α*PD-1 immunotherapy mediated by CD8+ T cells. IPA further enhances the anti-tumor immune response by increasing H3K27 acetylation in the Tcf7 super enhancer region, modulating the dry program of CD8+ T cells, and promoting the production of depletion precursor CD8+ T cells (Jia et al., 2024). Additionally, certain intestinal bacteria produce androgens, which can increase both the incidence of and resistance to therapy in prostate cancer (O'Leary, 2021a), in a mouse model, infection with *Helicobacter hepaticus* through the gastrointestinal tract led to a systemic elevation of pro-inflammatory cytokines, including TNF-α, IL-1α, IL-3, and eotaxin. These mice exhibited significant increases in precursors of prostate cancer, such as prostatic intraepithelial neoplasia and microadenocarcinoma (Poutahidis et al., 2013).

#### 2.2.3 Other aspects

The complex relationship between bacteria and cancer is not limited to the mouth and gastrointestinal tract, with new research showing that bacteria in other parts of the body are also strongly linked to the progression of cancer. For instance, microbial analysis of endometrium from patients with endometrial cancer and healthy volunteers revealed that *Prevotella* and *Pelomonas* were enriched in the endometrial cancer group, *Prevotella* was significantly associated with three genes (PRSS33, CPB2, XBP1). These genes are involved in the degradation of fibrin, the regulation of the coagulation and fibrinolysis system, and the regulation of the cell secretion system, thus promoting the progression of endometrial cancer (Li et al., 2021); bacteria associated with bacterial vaginosis, including *Lancefieldella parvula*, *Fusobacterium gonidiaformans*, *F. nucleatum*, *Peptoniphilus lacrimalis*, and *Porphyromonas uenonis* induces cervical cancer progression through direct cytotoxic effects on cervical cells, alterations in immune regulation, metabolic pathways, and interference with lipid metabolism (Maarsingh et al., 2022).

The progression of lung cancer is also linked to bacterial infections of the respiratory tract. According to the findings of Ye et al. (2016), gram-negative bacterial infection, particularly *Escherichia coli*, significantly enhances the expression of key lipid synthesis enzymes, FASN and ACC1, by activating TLR4 and TLR9 signaling pathways in NSCLC cells, this upregulation leads to increased lipid levels, thereby promoting the proliferation and metastatic potential of NSCLC cells. In NSCLC, significant alterations in the gut microbiota structure of mice have been observed. Specifically, the abundance of bacterial genera such as *Prevotella*, *Roseburia*, and *Gemmiger* increased markedly. Oral administration of *P. copri* to mice exacerbated inflammation, disrupted immune homeostasis, and led to significant structural changes in lung tissue, ultimately contributing to the progression and development of NSCLC (Qian et al., 2022).

The intricate relationship between bacteria and various cancers underscores the pivotal role of the microbiome in disease progression and treatment. These insights open new avenues for therapeutic strategies, such as the use of probiotics and the modulation of bacterial metabolites, to reduce cancer risk and improve treatment outcome. Understanding and manipulating the microbiome could be key to developing personalized cancer therapies in the future.

## 3 The therapeutic potential of bacteria in cancer

**Figure 2:**
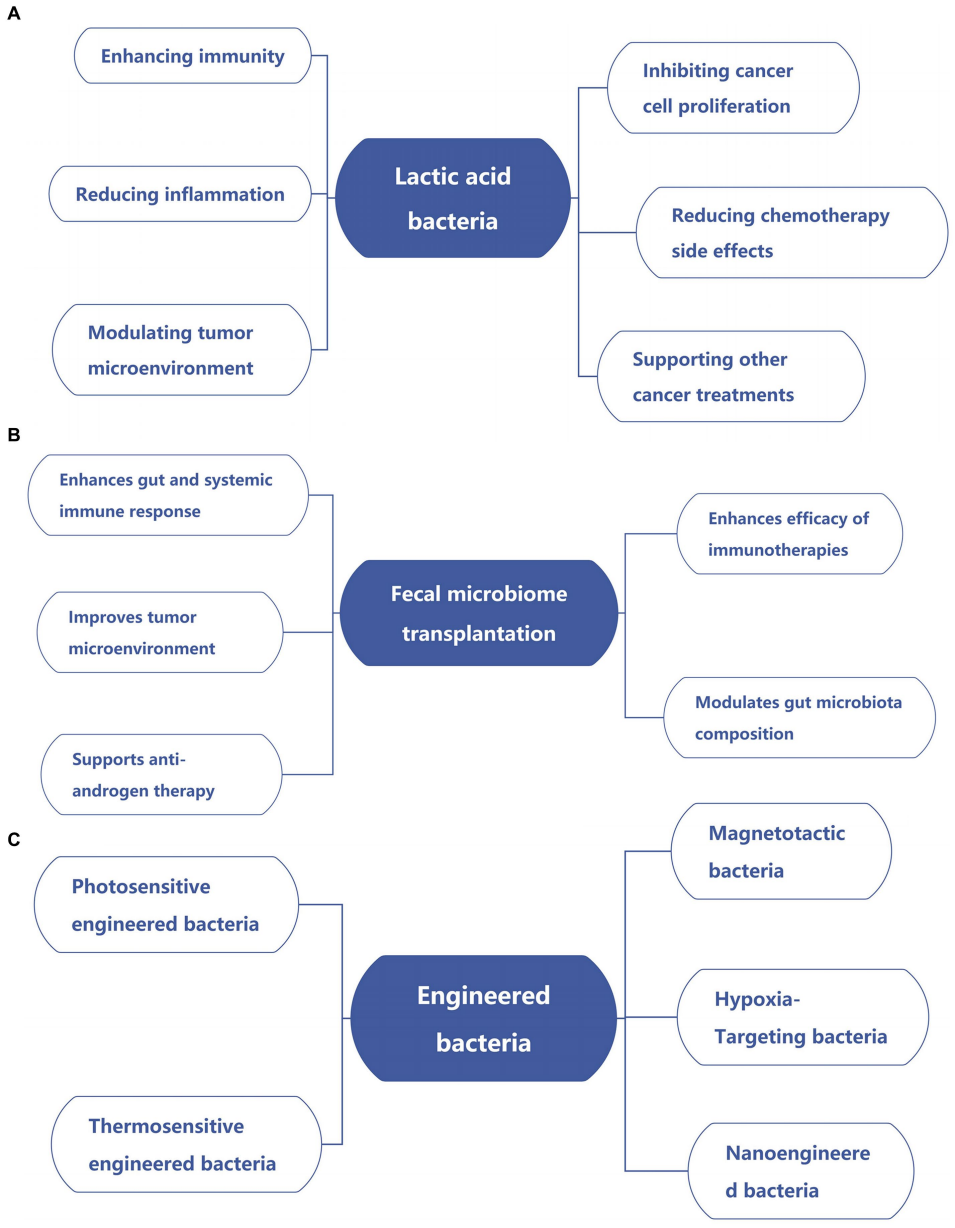
Emerging strategies for bacterial-based cancer therapies. **(A)** Lactic acid bacteria in cancer therapy. **(B)** Impact of fecal microbiome transplantation on cancer. **(C)** Applications of engineered bacteria in cancer.

As research progresses, the potential of bacteria to treat various cancers is increasingly being recognized. Here are the main ways to use bacteria to treat cancer (Figure 2; Table 3).

**Table 3:** Therapeutic effects of engineered bacteria in different types of cancer.

Strain(s)	Type of cancer	Bacterial action	References
*Clostridium* *Salmonella* *Bifidobacterium breve*	Solid tumor	Deliver therapeutic drugs directly to tumor cells	Tangney (2010), Fujimori (2008), LI et al. (2010)
Anaerobic *Bifidobacterium infantis*	Breast tumors	Transport of loaded nanoparticles into tumor cells	Xiao et al. (2022)
Non-invasive *Escherichia coli*	Plasmid transfection Genetically modify	Photodynamic therapy of tumors Carrying overexpressed genes or drugs to enhance anti-tumor effects	Deng et al. (2021)
Attenuated *Salmonella*	Solid tumor	Express and release the fluorescent protein ZsGreen, which is highly sensitive to the identification of small tumor tissue	Panteli et al. (2015)
*Bifidobacterium bifidum*	Hypoxic region of solid tumor	*Bifidobacterium bifidum* can be used to transfect plasmids and deliver semiconductor nanocrystals, plasmids transfecting cytosine deaminase genes can colonize hypoxic areas of solid tumors and produce cytosine deaminase, the transferred semiconductor nanocrystals can be used for tumor imaging or treatment	Min et al. (2008), Leschner and Weiss (2016)
*Rhodobacter sphaeroides* 2.4.1	Solid tumor	Emit near-infrared fluorescence, which aids in visualizing the interaction between bacteria and tumor tissue	Kwon et al. (2014)
*Escherichia coli* Nisle 1917 (EcN)	Solid tumor	Releasing flagellin B (flaB) and bind to lanthanide up-conversion nanoparticles (UCNP). UCNP emits light in the blue region, activating EcN to secrete flaB, and then it attaches to Toll-like receptor 5 present on macrophage membranes, triggering the immune response against tumor cells through the MyD88-dependent signaling pathway	Zhu et al. (2022)
Living photosynthetic bacteria (PSB)	Solid tumor	As targeted carriers for hypoxic tumor therapy	Zheng et al. (2021)
Thermal-sensitive engineering bacteria	Solid tumor	Stimulated by heat, they will produce tumor necrosis factor *α* (TNF-α) in the tumor to inhibit tumor growth Express programmed cell death protein (PD1) within the tumor tissue	Li et al. (2022)
Magnetotactic bacteria (MTB)	Anoxic zone of a tumor	Penetrate the interval of 3D multichannel tube sphere Inhibiting tumor cell growth through magnetotic bacteria’s magnetic field swing	Kotakadi et al. (2022), Kuzajewska et al. (2020), Mokrani et al. (2010), Wang et al. (2022), Ma et al. (2023)

### 3.1 Effect of probiotics on cancer

Lactic acid bacteria, a well-known group of probiotics, are gram-positive microorganisms essential for maintaining the stability of the gastrointestinal flora. These bacteria and their metabolites have been shown to enhance immunity, improve antioxidant capacity, and reduce blood sugar and cholesterol levels (Liu et al., 2021). Research suggests that culturing CAco2 cells with lactic acid bacteria in combination with 5-FU, and using this approach to treat breast cancer in mice, can mitigate the adverse effects of chemotherapeutic drugs on cells without compromising their anti-tumor efficacy (Levit et al., 2021). Lactic acid bacteria have been found to reduce Stat3 expression and secrete IL-6, thereby inhibiting breast cancer stem cells, suggesting their potential in breast cancer treatment and prevention (Dwi Ningtiyas et al., 2021; Choi et al., 2018; Ohashi et al., 2002).

Beyond breast cancer, lactic acid bacteria also play a significant role in the treatment and prevention of bladder cancer, regular consumption of lactic acid bacteria has been associated with a lower incidence of bladder cancer, likely due to their ability to modulate the immune response and maintain a healthy urinary tract microbiome (Ohashi et al., 2002).

In colorectal cancer, lactic acid bacteria have been shown to significantly enhance host immunity and reduce intestinal inflammation, they inhibit cancer cell proliferation by producing volatile fatty acids, adhering to tumor cells, and reducing harmful bacteria within tumor tissue. Notably, *Lactococcus lactis* has been shown to enrich the gut microbiota with beneficial probiotics and secrete the functional protein alpha-mannosidase, which exerts significant anti-tumor effects, this protein can inhibit the growth of colorectal cancer cells and patient-derived organoids in both *in vitro* and *in vivo* experiments, leading to reduced tumor volume in mouse models (Su et al., 2024).

In gastric cancer, lactic acid bacteria inhibit the growth of *Helicobacter pylori*—a major risk factor for gastric cancer—by producing organic acids and antimicrobial substances. This inhibition reduces the risk of chronic inflammation and malignant transformation of the gastric mucosa (Hwang et al., 2012). Furthermore, long-term consumption of probiotics has been associated with a reduction in tumor size and number, as well as an increase in IL-18 production. Studies have also shown that lactic acid bacteria extracted from Kefir can enhance the cytotoxic effects of human natural killer cells on chronic myeloid leukemia and colorectal cancer cells. This cytotoxicity is mediated through the modulation of the immune system, underscoring the potential of lactic acid bacteria to enhance the efficacy of immunotherapies (Yamane et al., 2018; Riaz Rajoka et al., 2017; del Carmen et al., 2017; Hradicka et al., 2020). However, the effectiveness of lactic acid bacteria can be influenced by the strains used, the dosage, and the duration of treatment. Differences in experimental design across studies make it challenging to compare results directly, and further standardization in methodologies is needed to validate these findings across different populations and conditions.

In summary, lactic acid bacteria contribute to cancer prevention and treatment across various types of cancer, by modulating immune responses, reducing inflammation, and inhibiting tumor growth through both direct and indirect mechanisms.

### 3.2 The therapeutic effect of fecal microbial transplantation on tumor

Fecal microbiome transplantation (FMT) has been gaining increasing attention as a strategy to enhance the efficacy of cancer treatment by regulating the gut microbiota, which can enhance the anti-tumor effect by reconstructing the gut microbiota and enhancing the gut and systemic immune response (Lu et al., 2022; Zaman et al., 2024; O'Leary, 2021b).

FMT has demonstrated significant therapeutic potential across various tumor types, including stomach cancer, colorectal cancer, melanoma, and liver cancer (Chen et al., 2023; Doocey et al., 2022; Wang et al., 2023). By reconstituting the gut microbiota in patients, FMT can enhance antigen presentation, promote effector T cell function, and improve both the tumor microenvironment and systemic immune responses, thereby increasing the effectiveness of immunotherapies such as PD-1 inhibitors. For instance, in melanoma patients who initially do not respond to PD-1 inhibitors, the combination of FMT and PD-1 inhibitors has been shown to induce partial or complete remission. Patients who respond favorably to this treatment tend to exhibit higher *α* diversity in their gut microbiota, with an enrichment of *Ruminococcaceae* and *Faecalibacterium,* These microbial communities enhance immune responses both systemically and within the tumor microenvironment by improving antigen presentation and effector T cell function (Baruch et al., 2021; Gopalakrishnan et al., 2018). Additionally, studies have found that FMT from healthy donors, when combined with PD-1 inhibitors such as nivolumab or pembrolizumab, significantly increases gut microbiota diversity and improves treatment outcomes in cancer patients, over time, the gut microbiota of responding patients becomes more similar to that of the donor, with an enrichment of immunogenic bacteria and a reduction in harmful bacteria following FMT. These changes enhance the efficacy of PD-1 inhibitors by strengthening both intestinal and systemic immune responses (Routy et al., 2023; Tanoue et al., 2019). Moreover, oral supplementation with *Akkermansia muciniphila* has been shown to restore the efficacy of PD-1 blockade by increasing the recruitment of specific T cells in tumor tissue. This effect is dependent on the IL-12 signaling pathway and is achieved through the recruitment of CCR9+CXCR3+CD4+ T lymphocytes within the tumor microenvironment (Routy et al., 2018). In the treatment of castration-resistant prostate cancer, changes in the gut microbiota are also closely related to the effect of anti-androgen therapy, and FMT shows potential in delaying castration-resistant prostate cancer (Pernigoni et al., 2021). For advanced renal cell carcinoma, the composition of the gut microbiota significantly influences the efficacy of immune checkpoint blocking (ICB). Antibiotics and tyrosine kinase inhibitors reduce the efficacy of opdivo by altering the gut microbiota, particularly by increasing the abundance of *Clostridium Hathewayi*. The therapeutic efficacy of ICBs can be improved through FMT or the introduction of beneficial symbiotic bacteria, further suggesting the potential for gut microbiome regulation as a strategy for enhancing cancer immunotherapy (Derosa et al., 2020). FMT has shown promise in enhancing cancer treatment, but the methodologies used in these studies warrant careful consideration. Variability in donor selection, preparation methods, and FMT administration routes can lead to inconsistent results. Additionally, the lack of standardized protocols across studies and potential biases related to donor microbiome composition highlight the need for more rigorous, large-scale clinical trials to validate the therapeutic benefits of FMT.

These clinical studies offer compelling evidence that modulating the gut microbiota can significantly enhance the effectiveness of cancer immunotherapy, particularly in patients who have not responded to conventional immune checkpoint inhibitors. These findings not only advance our understanding of the intricate interactions between microbes and the host immune system but also lay a crucial theoretical foundation for the development of novel biotherapeutic agents in the future.

### 3.3 Delivery, imaging, and targeted therapy of tumors

In tumor treatment, conventional therapies like radiotherapy and chemotherapy affect not only tumor cells but also healthy cells throughout the body, therefore, targeted therapies that specifically focus on tumor cells have significant potential. The tumor has a high retention and permeability effect on biologically compatible macromolecules such as liposomes, polymer-bound anticancer medicines, micelles and so on, intravenous bacterium injection has a similar effect on biological macromolecules (Fang et al., 2016), similarly, intravenous injection of bacteria can mimic this effect, making bacteria effective carriers for delivering therapeutic agents directly to tumor cells, such as C*lostridium*, S*almonella*, *Bifidobacterium breve*, etc. (Tangney, 2010; Fujimori, 2008; LI et al., 2010). Xiao et al. (2022) introduced a biocompatible bacterial/nanoparticle hybrid platform (Bif@DOX-NPs), utilizing anaerobic *Bifidobacterium infantis* to efficiently deliver doxorubicin-loaded bovine serum albumin nanoparticles directly into breast tumors; Deng et al. (2021) genetically modified non-invasive *Escherichia coli* through plasmid transfection to exhibit catalase activity, converting H2O2 to O2 at the tumor site. Under near-infrared (NIR) light, O2 transforms into cytotoxic 1O2, enabling the destruction of tumor cells. In addition to the bacteria themselves, extracellular vesicles secreted by bacteria have also shown promise in cancer treatment. For instance, after intraperitoneal injection in mice, these vesicles can activate the inflammasome signaling pathway, inducing the secretion of interleukin-1β (IL-1β). The increase in IL-1βpromotes the production of antigen-presenting cell precursors, thereby enhancing the immune response during tumor antigen delivery (Liu et al., 2024).

Bacteria also play a role in tumor detection. Monitoring the specific location and concentration of bacteria targeting tumor cells is essential for adjusting treatment plans and observing therapeutic effects. Current imaging techniques, such as CT scans, have limited sensitivity for early tumor detection and recurrence monitoring. Panteli et al. (2015) developed a new detection technique using an engineered attenuated *Salmonella* expressing and releasing the fluorescent protein ZsGreen, which can identify a tumor mass as small as 0.043 mm. Other studies have shown that *Bifidobacterium bifidum* can transfer semiconductor nanocrystals, specifically quantum dots (QDs), to the deep tissues of solid tumors. These QDs can be folate-bound to target tumor cells expressing the folate receptor, aiding in tumor imaging and treatment, certain bacteria can fluorescently image tumor tissue using bacterial luciferase (Lux), with light detected by a cooled charge-coupled device detector (Min et al., 2008; Leschner and Weiss, 2016). Kwon and colleagues discovered *Rhodobacter sphaeroides* 2.4.1, a novel tumor-targeting bacterial strain capable of emitting near-infrared fluorescence, aiding in visualizing bacterial interactions with tumor tissue (Kwon et al., 2014).

In the realm of targeted tumor therapy, the role of bacteria is indispensable, and engineered bacteria can further enhance therapeutic efficacy (Wu et al., 2019; Jiang et al., 2022). For example, *Escherichia coli* Nisle 1917 was designed to release flagellin B when bound to lanthanide up-conversion nanoparticles, which emit blue light to activate immune responses against tumor cells (Zhu et al., 2022); Living photosynthetic bacteria have been used as targeted carriers for hypoxic tumor therapy, leveraging their near-infrared chemotaxis and facultative aerobic traits (Zheng et al., 2021); Thermally-sensitive engineered bacteria can produce TNF-*α* upon heat stimulation to inhibit tumor growth (Li et al., 2022; Xu et al., 2022) and colleagues created thermally-induced bacteria, which can express programmed cell death protein 1 within the tumor tissue. When combined with laser irradiation, it can not only destroy tumor tissue but also ameliorate the immunosuppressive phenomena of the tumor microenvironment by boosting PD1 expression, biomineralizing gold nanopar-ticles (Wang et al., 2021) induce the expression of ClyA under near-infrared laser irradiation to kill tumor cells. Nanoparticles can also transport cationic antimicrobial peptides (Parchebafi et al., 2022) with anticancer and antibacterial actions into tumor cells. However, due to the harmful and adverse effects of these accumulating chemicals in the body, their therapeutic application is limited. Magnetotactic bacteria (MTB) are nanoorganisms that can be steered and propelled to the anoxic zone of a tumor using an external magnetic field (Kotakadi et al., 2022). Magnetosomes are magnetic nanoparticles synthesized by a few magnetotactic bacteria that have great potential in targeted cancer treatment (Kuzajewska et al., 2020). *In vitro* experiment proof that (Mokrani et al., 2010) MTB has the ability to penetrate the interior of 3D multicellular tumor sphere when subjected to directional magnetic field. Wang et al. (2022) created a tumor suppression method based on magnetotactic bacteria’s magnetic field swing, using RGD peptide modified MTB bacteria, tumor cells can be targeted and continuous magnetic oscillation applied to their surfaces to limit tumor growth. Ma and colleagues genetically engineered magnetotactic *Escherichia coli* bacteria to achieve tumor-specific drug release and immunotherapy under the control of an alternating magnetic field. These bacteria, equipped with a heat-sensitive promoter, enabled the controlled expression of genes that release anti-CD47 nanobodies within the tumor, thereby activating an immune response. This approach demonstrated significant therapeutic effects on both primary and distant tumors (Ma et al., 2023).

These findings underscore the broad prospects of bacterial applications in tumor treatment and detection. In the future, by further optimizing bacterial carriers and imaging technologies, it is expected that more precise and efficient tumor treatment and detection can be achieved, promoting the development of personalized medicine and bringing new hope to cancer patients.

## 4 Conclusion and future prospects

Recent advances in cancer research have increasingly highlighted the critical role of the microbiome in influencing tumor development and therapeutic outcomes. The complex interplay between microbial communities and the host’s biological systems has opened new avenues for understanding cancer biology, presenting bacteria as not only contributors to cancer progression but also as potential allies in the fight against it. As delve deeper into the relationship between bacteria and cancer, novel diagnostic and therapeutic strategies are emerging. First, the distributional changes of specific bacteria in different cancer patients present potential biomarkers for early detection. Studies have shown that the microbial communities in patients with oral cancer, colorectal cancer, and gastric cancer differ significantly from those in healthy individuals. These differences can be exploited for early screening through non-invasive methods such as saliva and fecal analysis, aiding in early cancer detection and intervention. Additionally, probiotics and their metabolites have demonstrated significant therapeutic potential in enhancing host immune function, regulating gut microbiota balance, and alleviating the side effects of chemotherapy. Probiotics not only bolster the immune response against tumors but also mitigate the toxic side effects of chemotherapeutic drugs, thereby improving patient quality of life and offering a promising adjunctive treatment approach. In addition to probiotics, FMT has shown significant therapeutic potential in the treatment of many types of tumors. By modulating the gut microbiome, FMT can enhance antigen presentation, promote effector T cell function, improve the tumor microenvironment and systemic immune response, thereby improving the efficacy of immunotherapies such as PD-1 inhibitors. Especially in patients who do not respond to traditional immune checkpoint inhibitors, the application of FMT May alter treatment response and significantly improve efficacy. However, the long-term effects and safety of FMT need to be confirmed by further research and need to be supported by larger clinical trials and data. Moreover, the use of engineered bacteria as drug carriers for targeted therapy is rapidly advancing. These bacteria can specifically target tumor tissues and release therapeutic agents within the tumor microenvironment, significantly enhancing treatment efficacy while minimizing damage to normal tissues. As bacterial carriers and imaging technologies continue to be optimized, the potential of bacteria in tumor-targeted therapy will further expand. Concurrently, the application of bacteria in cancer immunotherapy also shows immense promise. Certain bacteria can activate the host immune system, thereby enhancing the immune response against tumors, providing a foundation for the integration of bacteria with immunotherapy, and potentially becoming a vital component of next-generation cancer treatments. The application of bacteria in tumor detection also holds great promise. Engineered bacteria can target tumor tissues and emit detectable signals, thereby improving early tumor detection rates and providing new methods for monitoring treatment efficacy. The combination of these bacteria-based imaging technologies with existing imaging modalities is expected to achieve higher sensitivity and specificity in clinical practice, optimizing treatment decisions. In summary, the application of bacteria in cancer diagnosis, treatment, and monitoring offers broad prospects, paving the way for personalized cancer therapy and potentially playing a crucial role in clinical practice.

While research on the role of bacteria in cancer holds exciting potential, numerous challenges persist in further elucidating the relationship between microbes and tumors. Initially, studies investigating the impact of bacteria on tumor development often rely on observational data and microbiome analyses, which are vulnerable to variables such as reverse causation and confounding factors, including fluctuations in diet, lifestyle, and oral hygiene. Furthermore, the identification and quantification of bacteria within tumor tissues present technical challenges. Despite the efficacy of advanced techniques like 16S rDNA sequencing, FISH, and QPCR, issues such as sample contamination, difficulties in bacterial quantification, sequencing accuracy, and sample selection remain critical factors that can undermine the reliability of results. These technical obstacles can lead to inconsistent experimental outcomes and add complexity to the research process. Moreover, although studies have demonstrated associations between specific bacteria and certain cancer types, further validation is required before these bacteria can be reliably used as markers for early detection and diagnosis. Larger, more robust studies are necessary to confirm the accuracy of bacterial profiles and to assess the generalizability of these markers across diverse populations and cancer types. Additionally, while current research has begun to uncover connections between bacteria and cancer, the underlying causal mechanisms remain largely unexplored. Many studies are limited by small sample sizes and often focus on specific cancer types or populations, leading to heterogeneity in study design. Differences in demographic characteristics, sample sizes, and analytical methods further complicate the understanding of bacteria’s role in cancer. These challenges are exacerbated by the absence of standardized research protocols, underscoring the need for larger, multi-center studies to enhance the robustness of findings. Finally, significant hurdles remain in the clinical application of microbial therapies for cancer. Ensuring the long-term safety and standardization of FMT protocols is essential for broader clinical adoption. Equally important is the optimization of engineered bacteria design to ensure precise tumor targeting while minimizing systemic side effects. The successful implementation of microbial-based personalized therapies will require addressing technical, safety, and ethical considerations to ensure their effectiveness in clinical practice.

Future research should focus on the following areas: (1) Mechanistic studies on bacteria-tumor interactions: Understanding how bacteria influence tumorigenesis at the molecular and cellular levels will aid in developing new therapeutic strategies; (2) The role of bacteria in early cancer prediction: Identifying specific bacteria that can be used as predictive markers for different cancer types; (3) Application of engineered bacteria in cancer therapy: Through genetic engineering, enhancing the targeting and therapeutic efficacy of bacteria while minimizing their impact on healthy tissues; (4) The use of bacteria in tumor monitoring: Further optimizing bacterial labeling and imaging techniques to improve the sensitivity and specificity of early tumor detection; (5) Microbiome and personalized medicine: Studying the characteristics of the microbiome in different cancer patients to explore personalized treatment strategies utilizing probiotics, bacterial metabolites and FMT.

In conclusion, the intricate interactions between bacteria and cancer offer both significant challenges and exciting opportunities. A deeper understanding of these relationships could pave the way for innovative approaches to cancer prevention, diagnosis, and treatment. By integrating microbiome research into cancer biology, we May uncover new therapeutic targets and pathways, ultimately enhancing patient outcomes. Continued exploration in this field is crucial to fully harness the potential of bacteria in combating cancer, laying the groundwork for future breakthroughs in oncology.
